# Integrating Neural Circuits Controlling Female Sexual Behavior

**DOI:** 10.3389/fnsys.2017.00042

**Published:** 2017-06-08

**Authors:** Paul E. Micevych, Robert L. Meisel

**Affiliations:** ^1^Laboratory of Neuroendocrinology, Department of Neurobiology, David Geffen School of Medicine at University of California, Los AngelesLos Angeles, CA, United States; ^2^Brain Research Institute, University of California, Los AngelesLos Angeles, CA, United States; ^3^Department of Neuroscience, University of MinnesotaMinneapolis, MN, United States

**Keywords:** estrogen, progesterone, MOR, β-endorphin, dopamine, D1 receptors, dendritic spines, membrane estrogen receptor

## Abstract

The hypothalamus is most often associated with innate behaviors such as is hunger, thirst and sex. While the expression of these behaviors important for survival of the individual or the species is nested within the hypothalamus, the desire (i.e., motivation) for them is centered within the mesolimbic reward circuitry. In this review, we will use female sexual behavior as a model to examine the interaction of these circuits. We will examine the evidence for a hypothalamic circuit that regulates consummatory aspects of reproductive behavior, i.e., lordosis behavior, a measure of sexual receptivity that involves estradiol membrane-initiated signaling in the arcuate nucleus (ARH), activating β-endorphin projections to the medial preoptic nucleus (MPN), which in turn modulate ventromedial hypothalamic nucleus (VMH) activity—the common output from the hypothalamus. Estradiol modulates not only a series of neuropeptides, transmitters and receptors but induces dendritic spines that are for estrogenic induction of lordosis behavior. Simultaneously, in the nucleus accumbens of the mesolimbic system, the mating experience produces long term changes in dopamine signaling and structure. Sexual experience sensitizes the response of nucleus accumbens neurons to dopamine signaling through the induction of a long lasting early immediate gene. While estrogen alone increases spines in the ARH, sexual experience increases dendritic spine density in the nucleus accumbens. These two circuits appear to converge onto the medial preoptic area where there is a reciprocal influence of motivational circuits on consummatory behavior and *vice versa*. While it has not been formally demonstrated in the human, such circuitry is generally highly conserved and thus, understanding the anatomy, neurochemistry and physiology can provide useful insight into the motivation for sexual behavior and other innate behaviors in humans.

## Introduction

**Figure 1:**
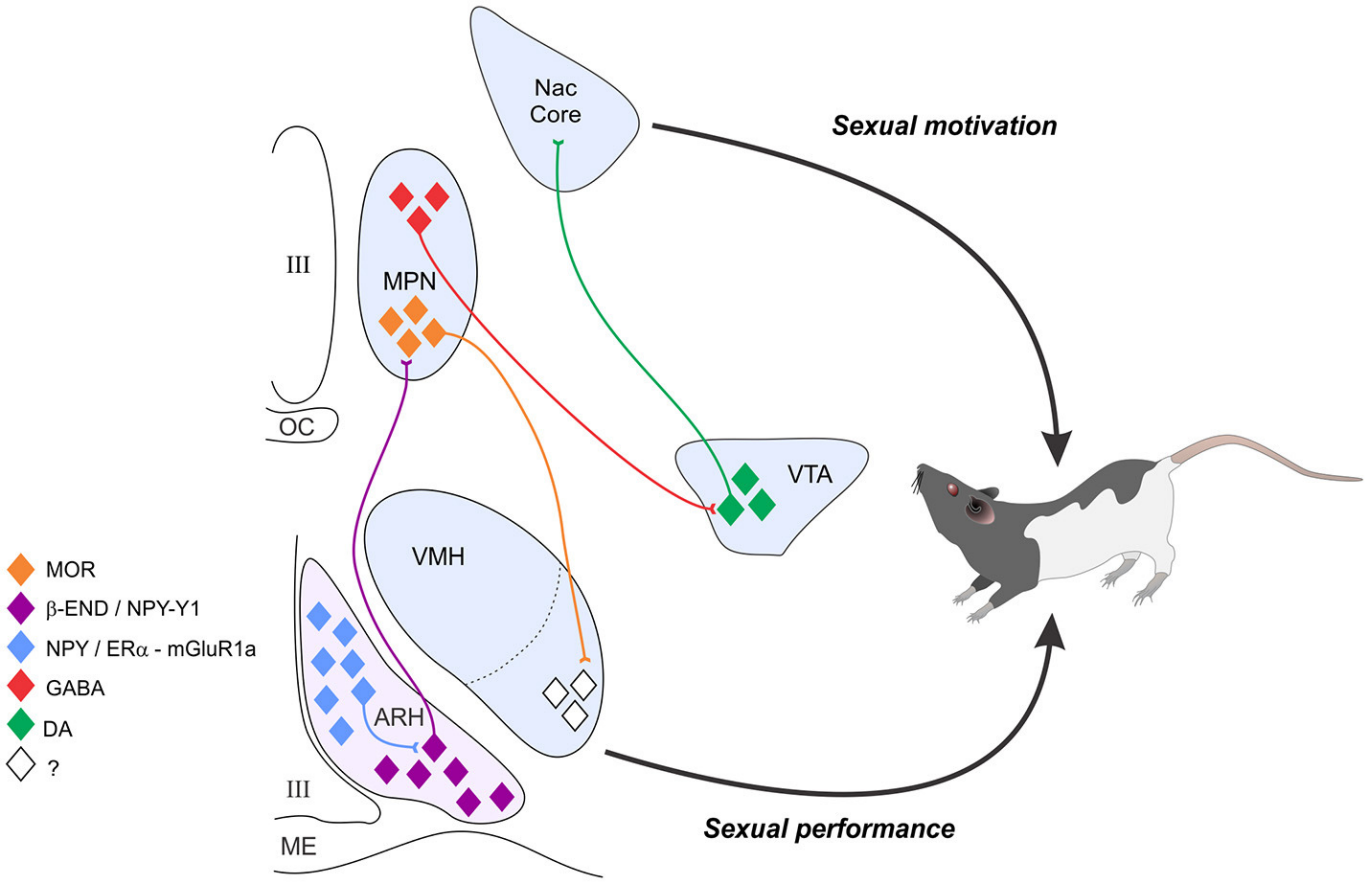
The estradiol induction of sexual receptivity in the female rat is indicated by lordosis behavior. The CNS regulation of this global response to hormonal and sensory input is regulated by a diffuse circuit that extends from the limbic system to the spinal cord. Within this lordosis regulating circuit, estradiol acts rapidly through estradiol membrane signaling (EMS) to release neuropeptide Y (NPY) in the arcuate nucleus of the hypothalamus (ARH), which activates β-endorphin (β-END) projection neurons that extend to the medial preoptic nucleus (MPN). The MPN is an important integrative node receiving accessory olfactory and limbic input. β-END activates MOR, producing a transient inhibition of the MPN which is relieved by progesterone in the cycling female. The MPN MOR neurons in turn project to the ventromedial nucleus of the hypothalamus (VMH), the final common output of the hypothalamus. The EMS and resulting transient inhibition is necessary for the full expression of lordosis behavior in the rat. In addition to its VMH efferents, the MPN sends a GABAergic inhibitory projection to the VTA. Estrogen inhibition of the MPN contributes to dopaminergic activation of the nucleus accumbens, which both regulates sexual motivation and mediates the rewarding consequences of sexual behavior. Estradiol's actions on the combined circuits serve to initiate sexual motivation in the male's presence, modulate the expression of sexual behavior to tactile stimulation provided by the mounting male, and to feed forward, increasing the efficiency of future copulatory interactions in a way that presumably increases reproductive success.

Mating, a social behavior, is directly influenced by hormonal state, which transmits information about the internal state of the animal to steroid responsive circuits in the nucleus accumbens and hypothalamus. These circuits integrate the hormonal state of the animal with environmental/sensory cues to produce an appropriate response (Micevych and Ulibarri, 1992). Female sexual behavior is divided into three components: attractivity, proceptivity, and receptivity (Beach, 1976). The best studied of these are proceptivity and receptivity. The behavioral manifestations of the motivation to copulate by a female are expressed as the female's willingness to accept the male's mount attempts and proceptive behaviors that include hopping, darting, and ear “wiggling” enticing the male to copulate. In the female rodent, developing ovarian follicles secrete estradiol into the peripheral circulation. This estradiol acts in mesolimbic circuits to increase motivation and in the hypothalamus to increase receptivity. Estradiol induces progesterone receptors (PR) within the hypothalamus (Parsons et al., 1979, 1980, 1981; McGinnis et al., 1981; Shughrue et al., 1997a; Alves et al., 2000) and stimulates synthesis of progesterone in astrocytes. This neuroprogesterone induces proceptive behaviors (Micevych and Sinchak, 2013), and the bolus of progesterone from the corpus luteum of the ovary acts on hypothalamic circuits to facilitate receptive behaviors since estradiol levels in the intact rat are insufficient to induce lordosis by themselves (Beach, 1948; Young, 1961; Powers, 1970; Sodersten and Eneroth, 1982). In addition to hypothalamic regions, motivation for mating behavior is heavily influenced by the release of dopamine (DA) in the nucleus accumbens. A persistent question in the field is the interaction of reward circuits and motoric circuits to elicit behavior, a model of which is summarized in Figure 1.

The mesolimbic motivational circuitry involves the classic reward circuit of projections from the ventral tegmental area (VTA) to the nucleus accumbens. Experiments in which the female rat paces the sexual encounter have demonstrated an increase in the release of dopamine in the reward pathway in anticipation of sexual contact (reviewed in Cummings and Becker, 2012) and female rats with basal forebrain damage including the nucleus accumbens resist the male's attempts to mount (e.g., Rivas and Mir, 1991).

The lordosis-regulating, receptive circuit involves limbic and hypothalamic nuclei including the posterodorsal medial amygdala (MeApd), the bed nucleus of the stria terminalis (BSt), medial preoptic nucleus (MPN), arcuate nucleus (ARH), and ventromedial nucleus (VMH) of the hypothalamus (Sinchak and Micevych, 2001; Sinchak et al., 2010; Polovin et al., 2012; reviewed in Micevych et al., 2015). It is in the ARH that estradiol has its initial behaviorally-relevant actions, which are mediated by membrane-initiated signaling. The VMH is the final common projection from the integrative hypothalamic and limbic circuits to the periaqueductal gray (PAG), reticular formation and vestibular nuclei, which in turn, send inputs to spinal motoneurons innervating trunk and neck musculature needed for the lordosis posture (reviewed in Pfaff et al., 1994)—a hallmark of sexual receptivity.

While, the common perception is that estradiol stimulates sexual receptivity, this is only the final result. The mechanisms in the interval between estradiol increasing in the blood and lordosis behavior are complicated and involve an inhibition in the hypothalamus but activation in the mesolimbic circuit. Ovariectomized (OVX) females are not sexually receptive with or without progesterone for approximately 24 h after estrogen (estradiol benzoate, EB) priming (Green et al., 1970; Quadagno et al., 1972; Sinchak and Micevych, 2001).

## Steroid activation of sexual receptivity

The ability to copulate with a male regardless of the her motivational state was defined by Beach as sexual receptivity, physically manifested as the lordosis reflex (Beach, 1948, 1976 and reviewed in Micevych and Sinchak, 2007). This posture includes a stereotypic arching of the back, elevation of the hindquarters, dorsiflexion of the tail and extension of the neck. The lordosis quotient, a measure of sexual receptivity, is defined as the number of lordosis reflexes by the female in response to the number of mounts X 100. As Sternson indicates, lordosis is among the most dramatic behavioral responses in neuroscience (Sternson, 2013). What makes the lordosis reflex so valuable, however, is its reproducibility (i.e., the lordosis quotient is repeatedly achieved for a specific dose of estradiol or estradiol + progesterone).

In the intact cycling rat, the sequential release of estrogens and progesterone from the ovary tightly regulates sexual receptivity. Various doses and timing of treatments with estradiol as well as treatments with estrogens and progesterone have been used over the years to induce the lordosis reflex (described in Micevych and Sinchak, 2013). What all of these paradigms have in common is that they all require estrogen and that sexual receptivity lags behind the administration of estradiol. This refractory period lasts ~20–24 h. Since the early 1980s, when it was demonstrated that gene transcription was needed for estrogenic induction of lordosis behavior, this lag-time was assumed to be due to the transcription of proteins necessary for lordosis behavior (Rainbow et al., 1980, 1982; Parsons et al., 1982). Among the first of these proteins to be discovered was PR (Parsons et al., 1979, 1980, 1981; McGinnis et al., 1981; Shughrue et al., 1997b; Alves et al., 2000). Indeed, progesterone was necessary to induce lordosis with low exogenous estradiol doses and at “early” (~24 h post estradiol) time points (Parsons et al., 1980; Sinchak and Micevych, 2001). What this theory hypothesis does not explain is how estrogens alone induce lordosis, which required higher estrogen doses and a longer interval between estrogen treatment and the behavior (Pfaff, 1970 reviewed in Clemens and Weaver, 1985). What has emerged is the idea that different behavioral circuits are activated by estradiol only treatment compared with estrogen and progesterone (reviewed in Sinchak et al., 2015). In the ARH, estradiol-only facilitation of lordosis reflex requires the activation of the opioid receptor, ORL-1, but estradiol + progesterone does not (Borgquist et al., 2014). While the motivation of sexual behavior is driven by the pleasure derived from the copulatory act (e.g., Meisel and Mullins, 2006), reproduction requires the coordination of sexual receptivity with the production of a viable oocyte that can be fertilized. As mentioned above, this stimulus is estradiol which rises during the estrous cycle until it peaks on proestrus. Circulating levels of (ovarian) progesterone are elevated several hours after the female becomes sexually receptive (Moss, 1974; Sodersten and Eneroth, 1981). We tested the idea that in the intact rat both estradiol and progesterone were needed for sexual receptivity by treating OVX/ADX rats with 10 μg EB and then 48 h later with 17 β-estradiol. In this paradigm blockade of progesterone receptors or steroidogenesis did not attenuate sexual receptivity, but did block proceptive behaviors (Micevych et al., 2008; Micevych and Sinchak, 2011). These results support the idea that lordosis is sensitive to estradiol levels and progesterone is responsible for inducing proceptive behaviors (Tennent et al., 1980; Lumia et al., 1981) and demonstrate that neuroprogesterone mediates proceptive behaviors. Further, the study showed that neither progesterone nor progesterone receptors are needed for estradiol-only induced lordosis. Finally, progesterone terminates lordosis behavior. Thus, progesterone's action are bi-phasic, first it augments the estradiol induced lordosis behavior and then prevents it (Goy et al., 1966; Nadler, 1970; Sodersten and Eneroth, 1982; Meisel and Sterner, 1990).

### ARH to MPN to VMH circuit

The ARH to MPN neural subcircuit provides an excellent opportunity to examine steroid signaling that regulates sexual receptivity. Within the ARH, a population of β-endorphin (β-END) expressing neurons inhibit lordosis by acting on the μ-opioid receptor (MOR; Cheung and Hammer, 1995; Torii et al., 1999; Mills et al., 2004). β-END is one of several posttranslational proteins expressed in proopiomelanocortin (POMC) neurons. While much attention, in terms of metabolic control, has been lavished on the POMC neurons that project to the periventricular nucleus (Jacobowitz and O'Donohue, 1978; Bell et al., 2000; Melnick et al., 2007), another POMC population regulates sexual behavior through its projection to the MPN (Jacobowitz and O'Donohue, 1978; Cheung and Hammer, 1995; Bell et al., 2000; Ibrahim et al., 2003; Mills et al., 2004; Melnick et al., 2007). Reproductively important POMC neurons appear to have a distinct morphology, and sensitivity to MOR agonists and ATP-sensitive potassium (KATP) channel modulators (Jacobowitz and O'Donohue, 1978; Cheung and Hammer, 1995; Bell et al., 2000; Ibrahim et al., 2003; Mills et al., 2004; Melnick et al., 2007). An acute effect of estradiol treatment in OVX rats is the activation and internalization of MOR in the MPN, leading to inhibition of lordosis behavior (Sirinathsinghji et al., 1986; Pfaus and Pfaff, 1992; Sinchak and Micevych, 2001; Sanathara et al., 2011). Reversal of MOR activation produces a facilitation of sexual receptivity (Eckersell et al., 1998; Sinchak and Micevych, 2001; Micevych et al., 2003; Mills et al., 2004; Sinchak et al., 2005; Dewing et al., 2007).

Throughout the estrous cycle, the pattern of MPN MOR activation/internalization tracks the sexual receptivity of the female, that is, MOR are deactivated (internalized) on the evening of proestrus when the rat is sexually receptive and reactivated on the morning of estrus when she is no longer receptive (Sinchak and Micevych, 2003; reviewed in Micevych and Sinchak, 2013; Sinchak et al., 2015).

A series of experiments sought to identify the ER mediating this transient inhibition. First, the MOR activation was not observed in ERαKO mice but was present–in ERβKO mice (Micevych and Sinchak, 2013). Second, in the ARH, a membrane impermeable estradiol-biotin conjugate induced MOR activation (Dewing et al., 2007) indicating a membrane ERα (mERα). Third, mERα forms a signaling complex with metabotropic glutamate receptor-1a (mGluR1a) (mERα-mGluR1a) that activates MOR (Dewing et al., 2007, 2008). Fourth, estradiol membrane-initiated signaling (EMS) through the mERα-mGluR1a complex activates PKCθ to induce internalization of MPN MOP and actively inhibit lordosis (Dewing et al., 2008). It is well established that estradiol activates the POMC from the ARH. Estradiol sensitive inputs appear to modulate POMC activity. In the rat, estradiol appears to act on NPY neurons, a subpopulation of which express ERα mRNA (Sar et al., 1990; Simonian et al., 1999). In N-38s immortalized hypothalamic neurons that express NPY, we have shown mERα, which mediates the estradiol activation of PKCθ, increases extracellular-signal regulated kinases 1/2 (ERK1/2) and intracellular free calcium (Micevych and Dominguez, 2009; Dominguez et al., 2013), results consistent with an estradiol-induced activation of NPY-Y1receptors on MPN-projecting POMC neurons, which inhibit lordosis behavior (Clark et al., 1985; Mills et al., 2004).

Estradiol controls the level of cell signaling in the ARH through modulation of levels of mERα. In primary cultured neurons, astrocytes, immortalized neurons and *in vivo*, estradiol treatment transiently increases trafficking of ER to the membrane (Bondar et al., 2009; Dominguez and Micevych, 2010). Plasma mER levels are determined by a balance of trafficking to the membrane, requiring ER palmitoylation and interaction with caveolin-1 (CAV1; Razandi et al., 2002; Meitzen et al., 2013), and internalization, requiring phosphorylation and β-arrestin-1 (Arrb1; Dominguez et al., 2009). Knockdown of CAV1 prevented the trafficking of ERα to the plasma membrane *in vivo*. Interestingly, ERαΔ4, a splice variant of ERα concentrated on the membrane in cultured cells from nervous tissue (Gorosito et al., 2008; Bondar et al., 2009; Dominguez and Micevych, 2010; Dominguez et al., 2013) was still trafficked to the plasma membrane (Christensen et al., 2011). To ascertain the role of β-arrestin-1 (Arrb1) in receptor dynamics, we studied ERα and ERαΔ in ARH tissue and N-38 neurons (Wong et al., 2015). As expected, Arrb1 was critical for ERα internalization following estradiol stimulation, but unexpectedly, trafficking of ERαΔ4 was also dependent on Arrb1. With siRNA, which reduced Arrb1 protein by 80%, membrane levels of ERαΔ4 were almost half of the control levels. Interestingly, previous studies have indicated that EMS requires ERα transactivation of mGluR1a, and that ERαΔ4 does not associate with mGluR1a (Bondar et al., 2009). The loss of ERK1/2 activation after Arrb1 siRNA indicates that Arrb1 helps organize the mER signaling machinery. Moreover, this loss of Arrb1-dependent signaling in the female ARH prevented EB induced lordosis behavior indicating that microcircuits in the ARH activated by estradiol need Arrb1 to function.

The microcircuits in the ARH mediating estradiol regulation of behavior are much more complex than indicated by the previous discussion. One indication of this is the role of GABA in this nucleus. GABA_B_ receptors mediate both initial and sustained estradiol–induced activation of β-END release into the MPN (Sinchak et al., 2013). Inhibition of GABA_B_ receptors in the ARH blocked estradiol-induced MPN MOR activation, which is needed for lordosis behavior, (Torii and Kubo, 1994; Torii et al., 1995, 1996, 1997, 1999). Knockdown of the GABA synthetic enzymes, GAD65 and GAD67, prevented facilitation of lordosis (McCarthy et al., 1994). Together these results indicate that estradiol-induced MOR activation is maintained at least in part by GABA_B_ signaling. Antagonizing GABA_B_ receptors 30 h after estradiol priming mimics the action of progesterone in this circuit (Sinchak and Wagner, 2012). The idea that progesterone acts through the silencing GABA_B_ receptors is intriguing (Micevych and Sinchak, 2013). Nevertheless, these data indicate that the neurochemistry of sexual receptivity is a far from settled science and will require further research to unravel.

### Mesolimbic circuitry and sexual motivation

In contrast to the hypothalamic circuitry which regulates the expression of sexual receptivity—lordosis, the mesolimbic circuitry regulates behavioral processes key to the motivational control of the expression of female sexual behavior (Salamone et al., 2016). Bindra (1969) offered one of the early neurobiological conceptualizations of motivation in which an organism's internal physiological state interacts with environmental stimuli that have intrinsic value to create the “central motive state.” This central motive state in turn induces postural adjustments (e.g., lordosis) and organized motor outputs (e.g., hopping and darting) that comprise the species typical actions related to each central motive state. Further, with experience motivated behaviors can be conditioned to increase the range of incentive stimuli and the animal's responses. Berridge (2007) refined this conceptualization of motivation to argue that mesolimbic dopamine primarily responds to incentive salience which increase the animal's “wanting” of rewarding stimuli. Though the literature on female sexual behavior is rather limited, it is notable that basal forebrain lesions of the mesolimbic pathway encompassing the nucleus accumbens increase the likelihood that a female rat will resist the male's mounting attempts or more rapidly escape the male (a decrease in “wanting” incentive stimuli), though if the male is able to forcibly apply tactile flank stimulation the lesioned female will exhibit the postural adjustments of the reflexive lordosis response (Dohanich and McEwen, 1986; Rivas and Mir, 1990, 1991; Guarraci et al., 2002). Such early studies formed the basis for conclusions that the mesolimbic system was associated with the incentive motivational properties of female sexual behavior. Our view of the female sexual behavior literature is that mesolimbic dopamine is involved in the rewarding consequences of sex (or “liking”) and incentive salience (“wanting”), both of which can be modified by the animal's experience.

The biological relevance of sexual motivation is seen in the modification of subtle sexual responses thought to impact reproductive success. Female rats display a series of approach and avoidance behaviors to males which regulate the temporal frequency of mounting attempts by the male (Bermant, 1961; Edmonds et al., 1972). Indeed, there is an optimal number of intromissions and periodicity of those intromissions needed to stimulate a set of neuroendocrine reflexes requisite for successful uterine implantation of embryos, though the specific sensory requirements differ among species. This pattern of vaginal stimulation was termed the “species vaginal code” by Diamond (1970) and typically is the optimal pattern of stimulation needed to produce a conditioned place preference (Paredes and Vazquez, 1999).

The highly active pattern of approach/avoidance regulates the receipt of intromission for female rats. Female hamsters have a very different sexual behavior pattern in that they are relatively immobile, maintaining the lordosis posture for up to 90% of the interaction with the male; seemingly the male determines the pacing of intromissions. However, the female hamster has subtle perineal movements that regulate the male's ability to achieve intromission (Noble, 1979, 1980). Anesthetizing the female hamster's perineum dramatically reduces intromissions, pointing to the female's control of the mating interaction (Noble, 1980).

Pacing of sexual interactions with the male is seen the first time female rats or hamsters are placed with a male. Still, sexual experience can modify these sexual interactions (Bradley et al., 2005; Meerts et al., 2014). In hamsters this change in copulatory efficiency was tested by giving females different levels of sexual experience and measuring the frequency of intromissions by the male. Giving female hamsters 6 weekly tests (but not 2 tests) for sexual behavior increased the percentage of the male's mounts that included intromission (termed “hit rate”) on the following test (Bradley et al., 2005; Hedges et al., 2009). This was true whether that seventh test was conducted 1 or 6 weeks after the last sexual experience test (Bradley et al., 2005). Thus, sexual experience seems to increase the female hamster's ability to regulate intromission by the male (in Berridge's term, increases “wanting”) and these effects of experience are persistent for at least several months without any further experience.

Direct tests of the rewarding (or “liking”) consequences of female sexual behavior have most commonly used a conditioned place preference paradigm (Oldenburger et al., 1992; Meisel and Joppa, 1994; Paredes and Alonso, 1997). Here females are allowed to freely explore an apparatus containing two unfamiliar chambers, which determines the degree to which the female has an initial preference for either of the chambers. The female is then sequestered in one chamber during mating and placed alone in the other chamber over a series of conditioning trials. After the conditioning trials, the female is placed back in the apparatus alone and allowed to explore as in the preconditioning session. Importantly, control conditions in which females are successively placed alone (without the addition of mating or other stimuli) in both chambers are used to establish that simply repeated exposure to the chambers does not produce a change from the initial preference. A significant increase in the time spent in the chamber in which sexual behavior occurred in the postconditioning test compared with the preconditioning test is operationally defined as evidence for sexual reward.

Two other rather clever behavioral approaches have strengthened our understanding of the motivational control of female sexual behavior. One approach utilizes a bilevel chamber that capitalizes on the speed advantage of female rats to avoid and escape the male's approaches (Mendelson and Pfaus, 1989). The idea here is that the female can quickly change levels of the apparatus to pace her copulatory interactions with a male rat. Pfaus performed factor analyses on a number of female behaviors in this apparatus and was able to validate the distinction between appetitive/motivational responses and copulatory responses in female rats (Pfaus et al., 1990). Becker took a different approach (Cummings and Becker, 2012) by designing an operant chamber in which the male and female rats were separated by a sliding door. The female had the capability to make a nose poke response for the door to open to get access to the male. The male was tethered in a compartment on the other side of the door, which permitted him the freedom to mate with the female, but not to leave the compartment. Computer interfaced video tracking software recorded the location of the female in the apparatus, through which the computer closed the door when the female returned to her original compartment. In this way the female could control the pacing of access to the male.

For both rats and hamsters, pacing of the male's intromissions depends on estradiol and progesterone with the mesolimbic circuitry one target of these hormone effects.

### Spinogenesis: A common feature of hypothalamic and mesolimbic circuitry

#### Estradiol induces dendritic spines in the ARH

At this point in time, it is well-established that estradiol regulates morphological plasticity in various parts of the brain (Matsumoto and Arai, 1979; Woolley and McEwen, 1993; Staffend et al., 2011; reviewed in Micevych and Christensen, 2012). In the VMH, the final common pathway out of the hypothalamus of information relevant to lordosis behavior, estradiol increased spine density and dendritic branching (Frankfurt et al., 1990; Meisel and Luttrell, 1990; Calizo and Flanagan-Cato, 2000, 2002; Madeira et al., 2001; Gonzalez-Burgos et al., 2015). Interestingly, estradiol also reduced the length of long primary dendrites that extend laterally out of the VMH the potential site of afferents from the MPN that are inhibited by β-END (Sinchak et al., 2010). These results suggest that as MOR inhibition wears off or is blocked with progesterone, excitatory afferents contact newly formed dendritic spines, activating VMH neurons.

In the ARH, estradiol-induced morphological plasticity was shown to be necessary for the induction of lordosis behavior (Christensen et al., 2011, 2012; Christensen and Micevych, 2012). Estradiol treatment increases dendritic spine density within 4 h, and it remains stable for 48 h. However, the composition of spines with different morphology changed. The early appearing spines were filopodial, morphology suggestive of immature, inactive and unstable spines (Christensen et al., 2011). Such filopodial spines are highly labile, rapidly appearing and disappearing during intense neural activity until they are stabilized by contacting an appropriate presynaptic partner (Parnass et al., 2000; Grutzendler et al., 2002; Trachtenberg et al., 2002). At approximately 24 h, a time point at which lordosis can be elicited by prior progesterone treatment, the population has more mushroom-shaped spines. Mushroom-shaped spines appear to be stable and functional, with receptors and anchoring proteins that allow for synaptic transmission. Stabilization of spines requires mature postsynaptic spines with receptors anchored at the postsynaptic specialization by scaffold proteins (Srivastava et al., 2008; reviewed in Srivastava and Penzes, 2011; Micevych and Christensen, 2012), and a presynaptic element for synaptic communication.

An actin scaffold underlies dendritic spines. Indeed, spine formation requires rearrangement of the underlying actin cytoskeleton. In the ARH, an increase in β-actin immunoreactivity is correlated an increase in spines demonstrated with Golgi staining (Christensen et al., 2011). Estrogenic regulation of spinogenesis was shown to involve ERα-mGluR1a signaling leading to modulation of actin dynamics through phosphorylation of molecules important for spine formation including cofilin, an actin depolymerizing factor (for review see Sarmiere and Bamburg, 2004; Hotulainen and Hoogenraad, 2010; Sanchez et al., 2012). Cofilin must be deactivated (phosphorylated) to allow the formation of filamentous actin and new spines (Bamburg, 1999; Meng et al., 2002). Estradiol, within an hour, induces cofilin phosphorylation which can be inhibited by mGluR1a antagonism (Christensen et al., 2011). Cytochalasin D, which prevents β-actin polymerization, abrogated both estradiol-induced spine formation and lordosis behavior (Christensen et al., 2011). It has been proposed that estradiol rapidly induces labile spines but another stimulus is needed to stabilize them (Srivastava et al., 2008; reviewed in Srivastava and Penzes, 2011). On-going experiments point to membrane-initiated estradiol regulation of pre- and post-synaptic proteins, suggesting that, for stability, newly formed spines associate with a presynaptic element (Rudolph et al., 2016).

#### Sexual experience effects on dendritic spines in nucleus accumbens

Morphological changes in the mesolimbic system are dependent on both estradiol availability and on sexual experience. Estradiol treatment of either female rats or hamsters *decreases* spine density on medium spiny neurons in the core of the nucleus accumbens (Staffend et al., 2011; Peterson et al., 2015). The assumption is that estradiol exerts these effects on dendritic spine plasticity through membrane estrogen receptor interactions with metabotropic glutamate receptors (Micevych and Mermelstein, 2008). Consistent with this hypothesis are observations that pre-exposure to an mGluR5 antagonist blocks the estradiol effects on dendritic spines (Peterson et al., 2015).

Sexual experience *increases* dendritic spine density in medium spiny neurons of female hamsters, particularly in the core of the nucleus accumbens (Staffend et al., 2014). Dendritic spines receive excitatory, largely glutamatergic, inputs and have different morphologies which are thought to reflect biophysical properties impacting excitability of the neurons (Tonnesen and Nagerl, 2016). The increase in dendritic spines following sexual experience in hamsters was primarily associated with a change in filopodial spines, which have “silent synapses.” Developmentally, silent synapses are enriched in glutamatergic NMDA receptors with an absence of AMPA receptors (Liao et al., 1999). In adulthood, silent synapses contain both NMDA and AMPA receptors, though with a preponderance of NMDA receptors (Huang et al., 2009). As a proxy for electrophysiological characterization of silent synapses, we measured AMPA and NMDA receptors in the nucleus accumbens of female hamsters following sexual experience. No changes in AMPA receptor gene expression or protein for either the GluA1 or GluA2 subunits were detected. Similarly, there were no changes in NMDA receptors measured by levels of the NR2B subunit, however, increased phosphorylation of tyr1472 of the NR2B subunit was observed. This specific phosphorylation site confers membrane stability to NR2B containing NMDA receptors (Chen and Roche, 2007), providing indirect evidence that female sexual experience increases NMDA-biased silent synapses in the nucleus accumbens. Clearly this idea needs to be confirmed electrophysiologically.

Dopaminergic projections from the ventral tegmentum synapse on nucleus accumbens medium spiny neurons that express either excitatory dopamine D1 receptors or inhibitory D2 receptors (Missale et al., 1998). In general, each of these neuronal phenotypes has a different pattern of efferent projections (Kupchik et al., 2015). Knowing the phenotype of medium spiny neurons affected by sexual experience can be informative for developing hypotheses about the functional consequences of these changes in dendritic spines. Not only were the effects of sexual experience restricted to the core of the nucleus accumbens, but changes in spines were localized to the D1 containing medium spiny neurons (Staffend et al., 2014). These anatomical observations link observations of plasticity in neural pathways associated with intrinsic fixed-action behavioral sequences (Kalueff et al., 2016) to the control of female sexual motivation. In this way dopamine neurotransmission may be the mediator of sexual motivation in females. As sexual experience produces changes in the motivational components of sexual behavior, these changes in behavior are paralleled by a corresponding change in neuronal plasticity.

#### Sexual behavior stimulates mesolimbic dopamine release

Analysis of the mesolimbic dopamine system's role in sexual motivation began with microdialysis measurements of extracellular dopamine levels during sexual behavior in female rats and hamsters. Dopamine release in the nucleus accumbens of female rats during sexual encounters is associated with the female's ability to pace the mating interactions with the male (Mermelstein and Becker, 1995; Becker et al., 2001; Jenkins and Becker, 2001, 2003). Similarly, for female hamsters, dopamine is elevated in the nucleus accumbens during sexual interactions (Meisel et al., 1993) Dopamine release in the female hamster's nucleus accumbens occurred during mating only if the male achieved intromission (Kohlert et al., 1997). To expand on these findings, we are now using fixed potential carbon fiber recording from the nucleus accumbens providing an ~1 s temporal resolution of dopamine transients, which allows time locking the dopamine signal to specific components of the female's sexual interaction with the male. There is a strong concordance between the peak of the dopamine transients and the female's receipt of intromission by the mounting male. Collectively these results indicate that intromission is a salient signal for activation of the nucleus accumbens during sexual behavior in females and that this dopamine release does not depend on prior experience.

Further analyses of hamsters have tested the idea that sexual experience can potentiate the mesolimbic response to sexual stimuli in females. Our work indicated that with 6 (but not 3) prior sexual interactions there was an augmented release of dopamine relative to that seen in inexperienced female hamsters (Kohlert and Meisel, 1999), paralleling the change in hit rate noted previously. This “sensitized” dopamine response in hamsters was confirmed by c-Fos analysis in which mating increased the number of labeled neurons in the core of the nucleus accumbens, with an even greater elevation of labeled neurons in females with prior sexual experience (Bradley and Meisel, 2001).

#### Plasticity in dopamine signaling

Female sexual experience increases dopamine release in the nucleus accumbens during sex, and that increased dopamine release leads to changes in neuronal morphology. This raises the question of how changes in dopamine-mediated intracellular signaling underlie structural and behavioral plasticities? Female sexual experience does not affect the levels of either D1 or D2 receptors in the nucleus accumbens, nor does it impact D1 or D2 receptor binding (Staffend et al., 2014), yet there must be an enhancement of dopamine receptor signaling since c-Fos production is sensitized. Stimulating dopamine D1 receptors produces a greater cAMP response in homogenates from the nucleus accumbens of sexually-experienced vs. inexperienced female hamsters (Bradley et al., 2004). Though both Gpp(NH)p (a non-hydrolyzable GTP analog) and forskolin (a direct activator of adenylyl cyclase) increased cAMP accumulation in a concentration-dependent manner, the absence of any further augmentation by sexual experience on cAMP accumulation suggested that sexual experience either impacted the coupling of dopamine D1 receptors to G-proteins or modulated other G-protein regulators (e.g., RGS or AGS proteins). The observation that dopamine D1 signaling depends on interactions with caveolin-1 raises the possibility that sexual experience affects dopamine signaling by modulating caveolin-1 expression (Kong et al., 2007).

Several signaling events downstream from cAMP are impacted by sexual experience in female hamsters, particularly elements of MAP kinase signaling. MAP kinase signaling is relevant in this context since activity in this pathway is associated with neuronal plasticity (Sweatt, 2001). Sexual behavior testing does not impact MAP kinase signaling, as measured by levels of ERK 1/2 either in its phosphorylated state or as total protein. However, in sexually experienced females there is a dramatic increase in phosphorylated ERK1/2 soon after a subsequent test for sexual behavior (Meisel and Mullins, 2006). Thus ERK 1/2 phosphorylation is sensitized by sexual experience. This response of ERK 1/2 to sexual behavior may mediate the observed increases in c-Fos expression.

One important clue to potential molecular mediators of sexual experience on the nucleus accumbens came from behavioral results showing that the increase in copulatory efficiency in sexual interactions with male hamsters was maintained for over a month without further sexual experience. ΔFosB tuned out to be a good candidate as the molecular mediator of this long-term behavioral plasticity. This truncated variant of FosB confers a remarkable level of resistance to proteasome degradation (Ulery et al., 2006; Ulery and Nestler, 2007). No changes in pan-FosB immunocytochemical labeling were detected in the nucleus accumbens following an acute sex behavior test, but again in sexually experienced female hamsters there was an increase in FosB labeling (Meisel and Mullins, 2006). Further, overexpression of ΔFosB in the accumbens facilitated conditioned place preference in female hamsters given only two condition sessions (Hedges et al., 2009). Overexpression of ΔFosB in female hamsters also increased the male's ability to achieve intromission (i.e., increased hit rate) over control females given only two prior sex tests. Overexpressing ΔJunD, the dominant negative binding partner of ΔFosB (Winstanley et al., 2007), blocked the induction of a conditioned place preference after the requisite conditioning trials (Been et al., 2013). Collectively, these studies demonstrate that ΔFosB is a key molecular nexus for the effects of sexual experience on the long-lasting changes in sexual reward and the efficiency of copulatory interactions with a male.

## Integrating the circuits

At the same time that the neural systems underlying the different components of reproduction in female rodents are separable, clearly these elements require integration for successful reproduction. One possibility is that the activation of ovulation, lordosis, and sexual motivation are simply temporally coincident. Alternatively, there are nodes through which the different circuits connect to execute this integration. The link between the hypothalamic and mesolimbic circuits historically has been rather mysterious, though recent work provides an intriguing (though currently untested) hypothesis that the MPOA could be a potential node for this integration (Coria-Avila et al., 2014). Dominguez (along with others) traced projections from the MPOA to the VTA (Tobiansky et al., 2013, 2016). They reported that the majority of these neurons were GABAergic, suggesting that the MPOA provided an inhibitory input to the VTA and in turn to the nucleus accumbens. This inhibitory control was revealed by the use of cocaine as a pharmacologic reinforcer, which increased the number of c-Fos stained neurons in the nucleus accumbens of MPOA lesioned animals and correspondingly produced a stronger conditioned place preference (Tobiansky et al., 2013).

An analysis of estrogen receptors provided a key extension of the research on the MPOA as an interface between the hypothalamus and mesolimbic dopamine system. Anatomically, the majority of the MPOA to VTA projecting neurons stained positively for either ERα (~70%) or GPER (~35%) (Tobiansky et al., 2016). The functional significance of these estrogen receptor containing neurons was demonstrated through intra-MPOA estradiol infusions which enhanced cocaine mediated dopamine release in the NAc (Tobiansky et al., 2016). These results support the idea that the MPOA is a source of inhibition to the mesolimbic dopamine system, which is released by estradiol acting on these GABAergic projection neurons.

Modulation of dopaminergic neurotransmission may be a mechanism through which estradiol modulates the inhibitory tone of the MPOA. Dopamine D1 receptors generally signal through excitatory G proteins, whereas D2 receptors are coupled to inhibitory signaling pathways (Nishi et al., 1989; Jaber et al., 1996). In this way regulating the balance of D1:D2 signaling can impact the level of excitation in MPOA neurons. The results of immunocytochemical staining, Western blot analyses and autoradiographic receptor binding converged on the conclusion that estradiol biased the ratio toward D2 signaling, presumably reducing the intrinsic excitability of MPOA neurons (Graham et al., 2015). The functional impact of this altered dopaminergic signaling balance was mirrored by pharmacological analysis. Amphetamine (which would stimulate both D1 and D2 dopamine receptors) infused into the MPOA increased the amount of time before the female rat returned to the male following mounts and ejaculation (Guarraci et al., 2008). Direct MPOA infusion of a dopamine D2 agonist increased while a dopamine D1 agonist reduced sexual motivation measured in bilevel chambers (Graham and Pfaus, 2010), indicating bidirectional effects of dopamine receptor subtypes on sexual motivation. Collectively these results identify dopaminergic efferents as a potential source of estradiol modulation of MPOA inputs to the VTA.

The desire to engage in sexual behavior and the performance of sexual behavior are both neurally and functionally separable (Georgiadis et al., 2012). The MPOA is both anatomically and functionally positioned to integrate the actions of estradiol on sexual motivation through the mesolimbic system, as well as on the overt expression of lordosis through hypothalamic circuitry. At the same time, continuing research on the hypothalamic and mesolimbic systems controlling female sexual behavior will undoubtedly develop a more detailed understanding of how these anatomical and functional circuits are integrated.

## Author contributions

PM and RM each contributed background for this review.

### Conflict of interest statement

The authors declare that the research was conducted in the absence of any commercial or financial relationships that could be construed as a potential conflict of interest.
